# Functional Characterisation of Anticancer Activity in the Aqueous Extract of *Helicteres angustifolia* L. Roots

**DOI:** 10.1371/journal.pone.0152017

**Published:** 2016-03-24

**Authors:** Kejuan Li, Yue Yu, Shuang Sun, Ye Liu, Sukant Garg, Sunil C. Kaul, Zhongfang Lei, Ran Gao, Renu Wadhwa, Zhenya Zhang

**Affiliations:** 1 Graduate School of Life and Environmental Sciences, University of Tsukuba, 1-1-1 Tennodai, Tsukuba 305–8572, Japan; 2 Drug Discovery and Assets Innovation Laboratory, DAILAB, National Institute of Advanced Industrial Science & Technology (AIST), Central 5–41, 1-1-1 Higashi, Tsukuba 305–8565, Japan; 3 School of Integrative and Global Majors, University of Tsukuba, 1-1-1 Tennodai, Tsukuba 305–8577, Japan; 4 Institute of Laboratory Animal Sciences, Chinese Academy of Medical Sciences, Beijing 100021, China; Indian Institute of Technology (IIT) Delhi, INDIA

## Abstract

*Helicteres angustifolia* L. is a shrub that forms a common ingredient of several cancer treatment recipes in traditional medicine system both in China and Laos. In order to investigate molecular mechanisms of its anticancer activity, we prepared aqueous extract of *H**elicteres* *a**ngustifolia* L. Roots (AQHAR) and performed several *in vitro* assays using human normal fibroblasts (TIG-3) and osteosarcoma (U2OS). We found that AQHAR caused growth arrest/apoptosis of U2OS cells in a dose-dependent manner. It showed no cytotoxicity to TIG-3 cells at doses up to 50 μg/ml. Biochemical, imaging and cell cycle analyses revealed that it induces ROS signaling and DNA damage response selectively in cancer cells. The latter showed upregulation of p53, p21 and downregulation of Cyclin B1 and phospho-Rb. Furthermore, AQHAR-induced apoptosis was mediated by increase in pro-apoptotic proteins including cleaved PARP, caspases and Bax. Anti-apoptotic protein Bcl-2 showed decrease in AQHAR-treated U2OS cells. *In vivo* xenograft tumor assays in nude mice revealed dose-dependent suppression of tumor growth and lung metastasis with no toxicity to the animals suggesting that AQHAR could be a potent and safe natural drug for cancer treatment.

## Introduction

Cancer is a group of diseases involving abnormal cell growth that often acquire potential to invade or spread to other parts of the body. According to WHO, cancers rank among the leading causes of morbidity and mortality worldwide, with approximately 14 million new cases and 8.2 million cancer related deaths accounting for 14.6% of all human deaths in 2012. The number of new cases is expected to rise by about 70% over the next two decades [1, 2]. Surgery, radiotherapy and chemotherapy are the current mainstream cancer treatments. However, the prevalent chemotherapeutic drugs have a number of limitations, such as adverse side effects, limited efficacy, and high rate of secondary failures. Therefore, the research and development of anticancer drugs with high efficiency and low toxicity, particularly the drugs extracted from natural resources, is of great importance and has been attracting attention [3].

*Helicteres angustifolia* L. (*H*. *angustifolia*) is a small shrub widely distributed in slopping grassland such as the Southeast China, Laos, Japan, Australia and many Southeast countries. The dry root of *H*. *angustifolia*, known as “Shan Zhi Ma” in Chinese, has been popularly consumed as a medicinal liquor or tea to promote general wellness, and widely used to treat a variety of ailments ranging from influenza fever, carbuncle, parotitis, inflammation and cancer [4, 5]. Although popularly used for health benefits and therapeutic potentials, the scientific evidence to such activities is scarce. Previous investigations on active phytochemicals in the roots of *H*. *angustifolia* have revealed the identification of flavonoids [6], quinines [7], triterpenoids [8], cucurbitacins and their derivatives [9] in the chloroform and ethanol extracts that exhibited anti-proliferation activity in a variety of human cancer (colorectal- COLO 205 & HT-29, gastric- AGS, and ovarian- OVCA429, hepatic- HepG2 & BEL-7402 and melanoma- SK-MEL-28) cells [8, 10, 11]. Considering that the water extract is more favorable for human consumption, we investigated anticancer potential of aqueous extract of *H*. *angustifolia* roots (AQHAR) and found, for the first time, that it possesses potent anti-cancer activity [12]. In the present study, we demonstrate the mechanism, at least in part, of such anticancer activity using *in vitro* cell-based assays. Furthermore, its relevance to *in vivo* tumor suppression was determined using nude mice xenograft models.

## Results and Discussion

### Anti-proliferative effect of AQHAR on the growth of human osteosarcoma (U2OS) and normal fibroblasts (TIG-3)

**Fig 1:**
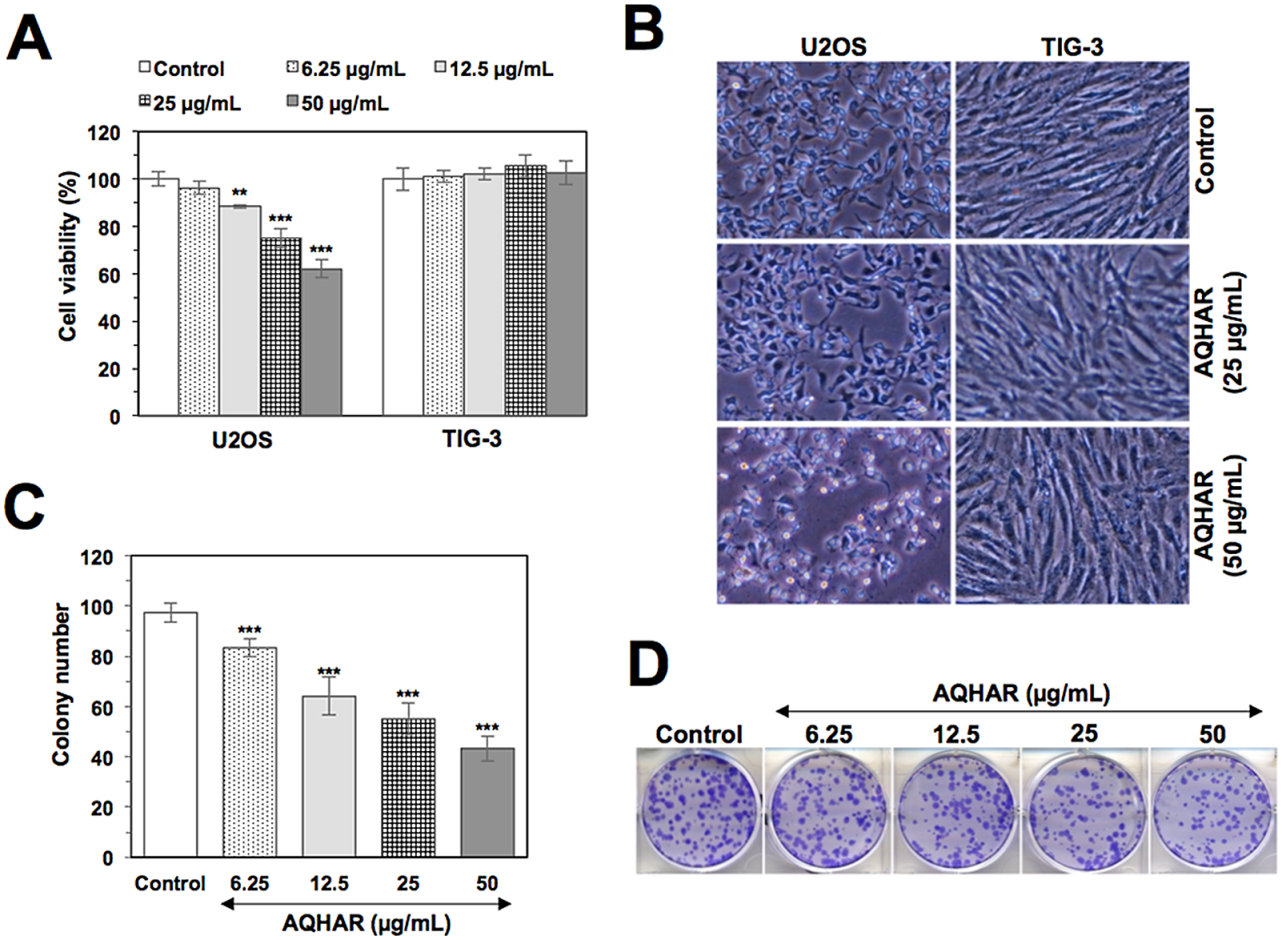
Selective cytotoxicity of AQHAR in human cancer cells. (A) Cell viability of human cancer and normal cells treated with indicated doses of AQHAR. Human osteosarcoma (U2OS) cells showed dose-dependent decrease in viability; normal fibroblasts (TIG-3) were not affected by the equivalent doses (B) Phase contrast images of human cancer and normal cells showing toxicity of AQHAR (50 μg/ml) to cancer cells only. (C and D) Dose dependent reduction in colony forming efficacy of AQHAR-treated U2OS cells. Results are represented as mean ± SD of three independent experiments. ***p<0.001 denotes statistically significance difference between the control and treated groups. AQHAR, aqueous extract of *Helicteres angustifolia* L. root.

Cytotoxicity of AQHAR on human cancer (osteosarcoma, U2OS) and normal fibroblasts (TIG-3) was examined by MTT assay. As shown in Fig 1A, AQHAR (50 μg/mL) inhibited U2OS cell proliferation in a dose-dependent manner with a cell viability of 62.13 ± 3.94%, while normal cells TIG-3 remained unaffected. The data was also evident by examining the morphology of cells under the microscope (Fig 1B), suggesting that AQHAR is selectively toxic to human cancer cells.

We evaluated the long-term effect of AQHAR on cell proliferation by colony formation assays. U2OS cells treated with AQHAR for 6 h showed inhibition of their colony-forming efficacy during 10 days of culture with regular change of normal medium, every three days. As shown in Fig 1C and 1D, colony number and size decreased significantly (*p*<0.001) when cells were treated with AQHAR (6.25, 12.5, 25, and 50 μg/mL).

### AQHAR induced G2/M cell cycle arrest and apoptosis in U2OS cells

**Fig 2:**
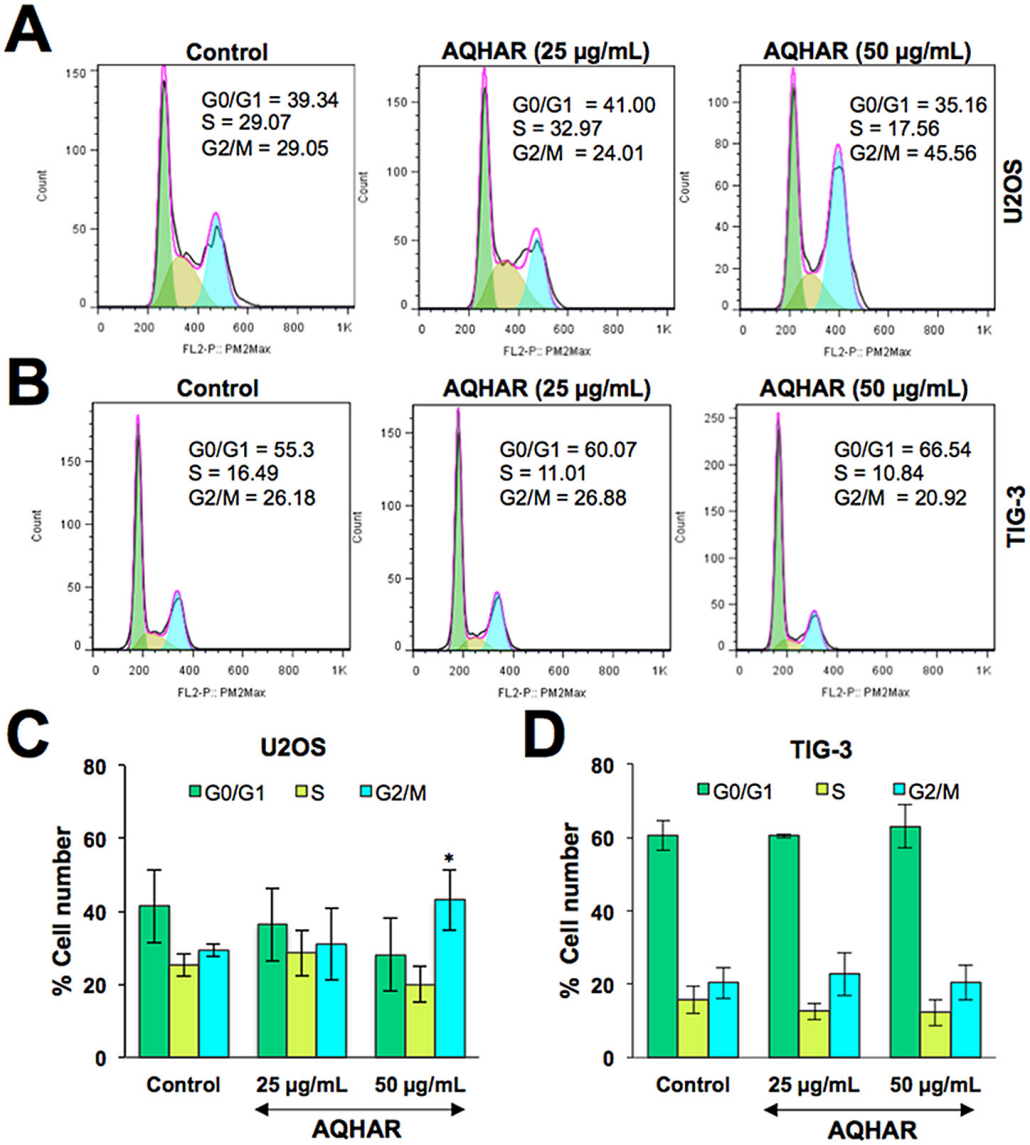
Cell cycle analysis of control and AQHAR treated cancer and normal human cells. Cell cycle distribution of control and AQHAR-treated U2OS (A and C) and TIG-3 (B and D) cells are shown. Results from three independent experiments are represented as mean ± SD. *p<0.05 denotes statistically significance difference between the control and treated groups.

Based on the above data, we investigated the effect of AQHAR on cell cycle progression of human cancer and normal cells. As shown in Fig 2A and 2C, the percentage of U2OS cells in G2/M phase increased from 29.29 ± 1.64% in control to 31.06 ± 9.96% and 43.16 ± 8.32% in 25 and 50 μg/mL of AQHAR-treated cells, respectively. The increase in cell population in G2 stage was accompanied by a decrease in number of cells in S phase. Of note, we found no change in cell cycle distribution in AQHAR-treated normal human fibroblasts (Fig 2B and 2D). The data was consistent with the selective cytotoxicity of AQHAR to cancer cells. Since such selective induction of growth arrest in cancer cells is believed to be extremely useful for cancer therapy [13–15], we next extended the analysis on cell cycle regulatory proteins.

We performed a flow cytometric analysis to evaluate healthy (Annexin V-/7- ADD-), early apoptotic (Annexin V+/7-ADD-), late apoptotic (Annexin V+/7-ADD+) and debris (Annexin V-/7-ADD+) cell populations in control and AQHAR-treated cells. Annexin V positive (early and late apoptosis) cells were considered as the apoptotic population.

**Fig 3:**
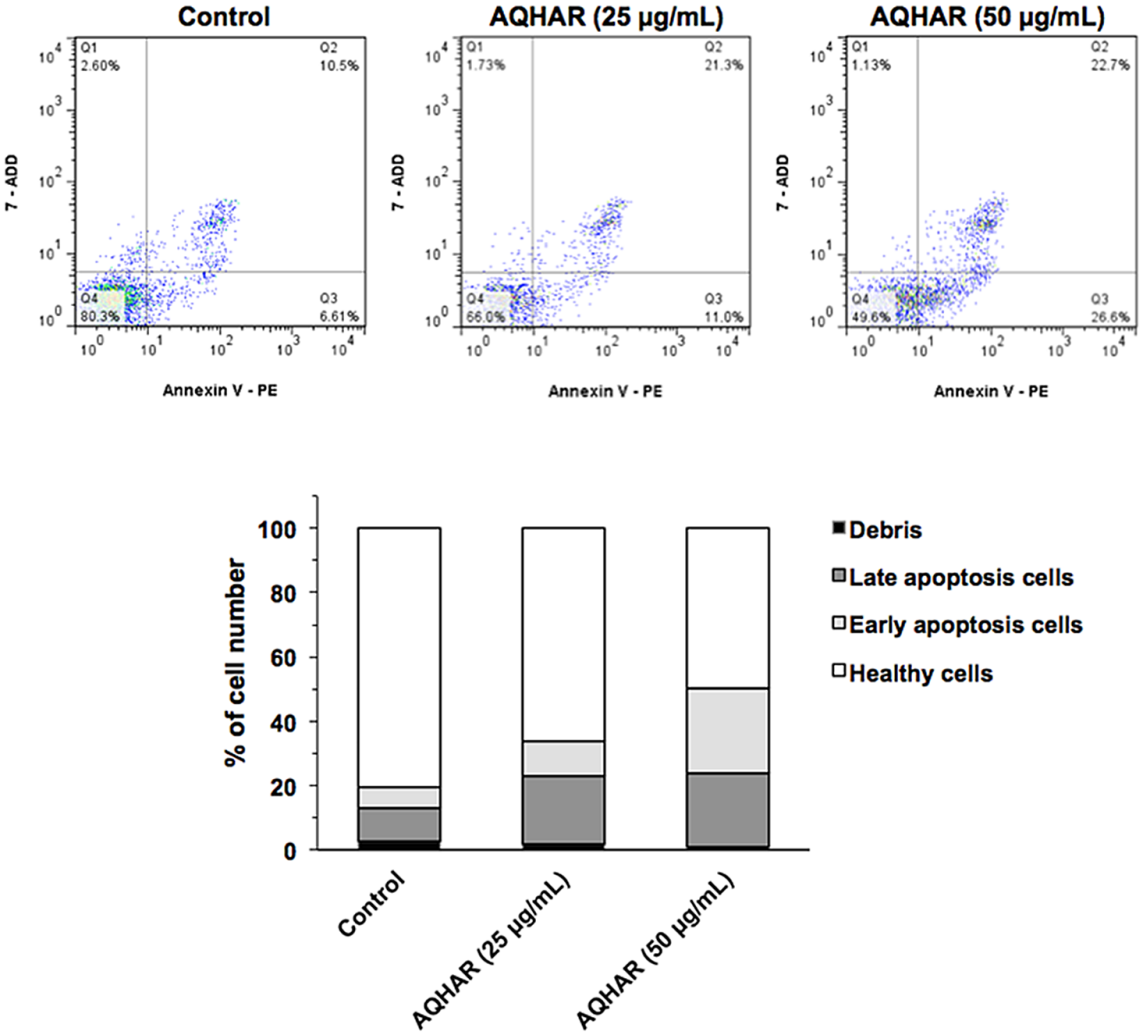
Induction of apoptosis by AQHAR in U2OS cells. Control and AQHAR-treated cell populations were examined for Annexin-V expression by cytometric analysis. As shown, increase in apoptotic cells was observed in cultures treated with increasing doses of AQHAR.

As shown in Fig 3, apoptotic population of U2OS cells increased significantly with increasing doses of AQHAR; 32.3% (with 25 μg/mL) and 49.3% (with 50 μg/mL), compared with the untreated control group (17.11%). Induction of apoptosis has been recognized as an efficient strategy for cancer chemotherapy and a useful indicator for cancer treatment and prevention [16]. In view of the present data, we extended the analysis to explore the effect of AQHAR on molecular markers of growth arrest and apoptosis.

### AQHAR-induced cell cycle arrest and apoptosis in cancer cells is mediated by activation of p53-p21 and caspase signaling

**Fig 4:**
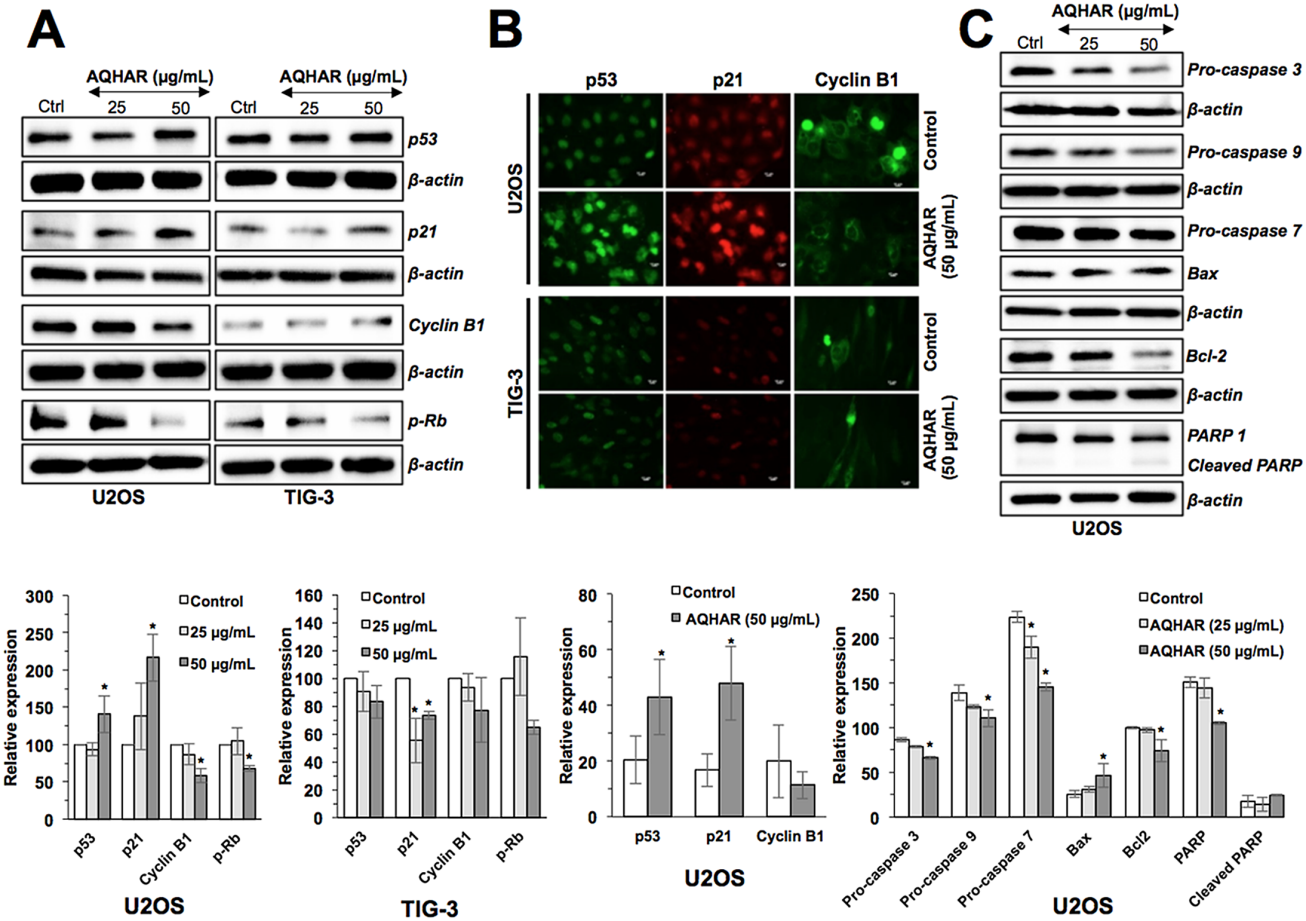
Effect of AQHAR on key regulators of cell cycle arrest and apoptosis in U2OS and TIG-3 cells. Cells were treated with AQHAR for 24–48 h and subjected to (A) Western blotting and (B) immunostaining for cell cycle regulatory proteins (p53, p21, Cyclin B1 and p-Rb). (C) Western blot analysis revealed activation of apoptotic signaling in U2OS cells. Quantitation of the results from three independent experiments is shown as mean ± SD with statistical significance as *p<0.05 between the control and AQHAR-treated cells.

In order to understand the mechanisms of AQHAR-induced G2/M arrest, we examined G2/M phase related regulatory proteins. As shown in Fig 4A, AQHAR (50 μg/mL) caused upregulation of p53 expression. p21, a p53-downstream effector involved in growth arrest, also showed increase in expression. Normal cells (TIG-3), however, showed no significant difference in p53 expression rather a minor decrease in p21 expression was observed as compared to untreated cells. These results were also confirmed by immunostaining of control and AQHAR-treated cells (Fig 4B). In parallel, we examined the level of G2/M arrest checkpoint protein, Cyclin B1. A dose-dependent decrease in Cyclin B1 was found in U2OS cells treated with AQHAR, while TIG-3 showed no significant difference (*p*<0.05) in control and treated cells. Immunostaining also confirmed the down regulation of Cyclin B1 in AQHAR-treated U2OS cells, and not in TIG-3, cells (Fig 4B). We also examined the expression of pRb by Western blotting (Fig 4A). Whereas the treatment with AQHAR (50 μg/mL) showed decrease in the expression of phosphorylated pRb in cancer cells, there was no significant difference in normal cells. Taken together, these results suggested that the selective cytotoxicity of AQHAR in U2OS cells was mediated by activation of p53 and pRb pathways.

In order to determine the molecular mechanism of AQHAR-induced apoptosis, we examined major regulators of apoptotic signaling. Consistent with the flow-cytometric analysis, there was a sharp decrease in the expression of apoptosis-related proteins pro-caspase 3, pro-caspase 7, pro-caspase 9, Bcl-2, and PARP-1 and increase in Bax and cleaved PARP in a dose-dependent manner (Fig 4C). Taken together, the above data suggested that AQHAR activates two signaling pathways leading to growth arrest and apoptosis in cancer cells.

### AQHAR caused induction of DNA damage and ROS signaling in cancer cells

**Fig 5:**
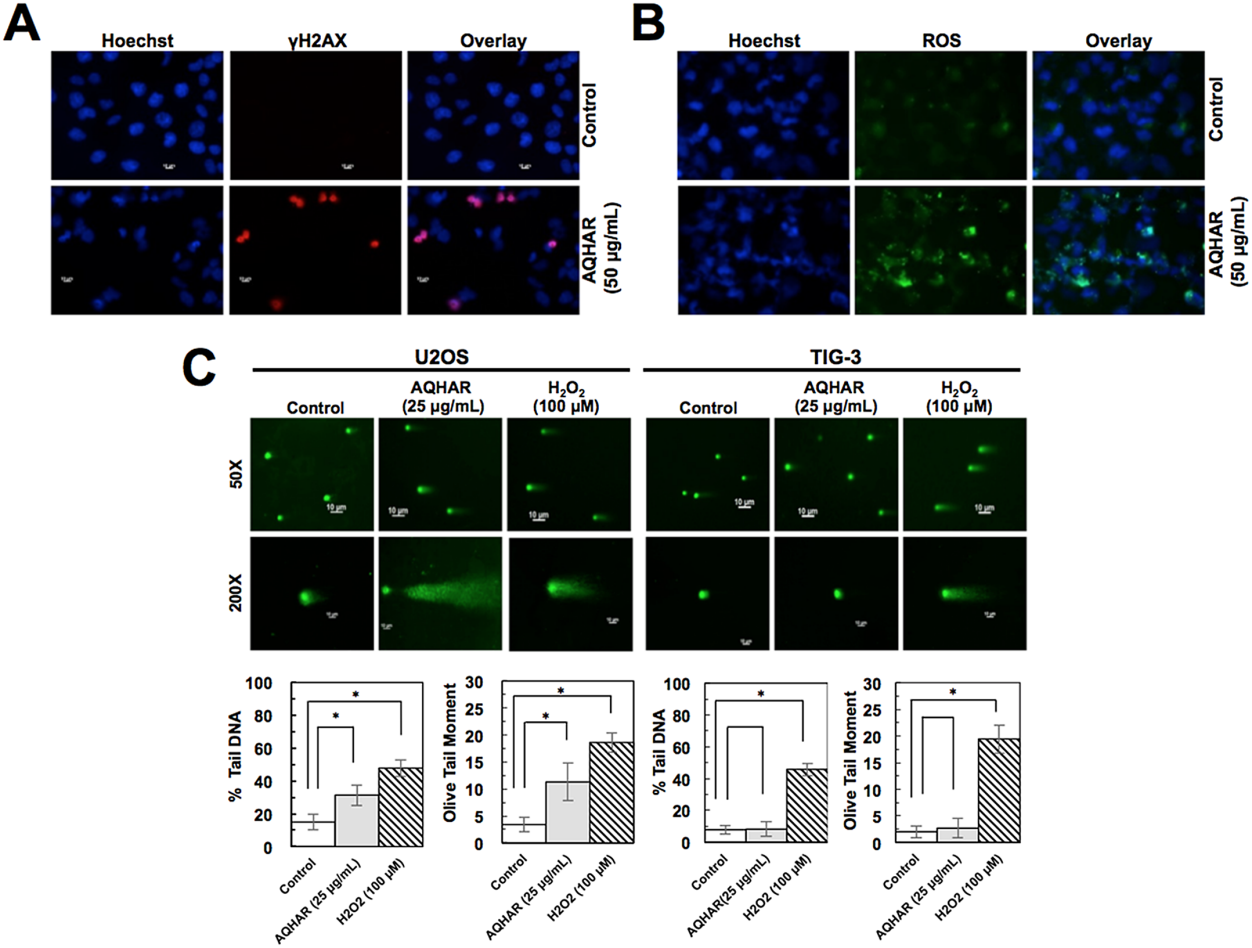
Induction of DNA damage and ROS by AQHAR treatment. AQHAR-treated cells exhibited increase in the γH2AX foci, signifying induction of DNA damage (A) and ROS (B) in U2OS cells. (C) Neutral comet assay in control and AQHAR-treated U2OS and TIG-3 cells. Mean percent Tail DNA and Tail moment was calculated using CASP Software. *p<0.05 denotes statistically significant difference between the control and treated U2OS cells. H_2_O_2_ (100 μM) was used as a positive control.

Based on the above results that the treament with AQHAR induces growth arrest and apoptosis, we hypothesized that it might be mediated by activation of DNA damage signaling. Control and AQHAR-treated cells were examined for γH2AX, a sensitive and reliable marker for DNA double-strand breaks [17] and activation of DNA damage response. Immunostaining results revealed an increase in γH2AX (Fig 5A) and ROS (Fig 5B) in response to treatment with AQHAR (50 μg/mL) compared to untreated (control) U2OS cells. We also performed comet assay. U2OS cells treated with 25 μg/mL of AQHAR for 24 h or 100 μM of H_2_O_2_ for 20 min (positive control) were collected for comet assay. As shown in Fig 5C, compared with the untreated control, AQHAR (25 μg/mL) induced DNA damage in U2OS cells, as indicated by the presence of DNA tail. Normal cells (TIG-3) showed no DNA tail after AQHAR treatment, whereas a larger area and longer DNA tail length reflected extensive DNA damage in cells treated with H_2_O_2_ (100 μM). DNA damage was also characterized as the amount of DNA in “comet” tail (% Tail DNA) and its combination with distance of migration (Olive tail moment) [18, 19]. As shown in Fig 5C, the percentage of total cell DNA in the tail significantly (*p*<0.05) increased from 14.94 ± 4.82% (control) to 31.36 ± 6.35% (25 μg/mL of AQHAR), and the Olive tail moment from 3.39 ± 1.32% (control) to 11.29 ± 3.45% in response to treatment with 25 μg/mL of AQHAR, respectively. TIG-3 cells treated with the same concentration showed no significant difference (*p*<0.05) as compared to those untreated cells. These results suggest that AQHAR inhibits cell proliferation by activating DNA damge response in cancer cells; normal cells remain unaffected.

We next investigated the involvement of stress and DNA damage signaling in response to AQHAR treatment in U2OS cells. As shown in Fig 5C, an induction of ROS was detected in AQHAR-treated U2OS cells. ROS are chemically reactive molecules that have essential role in signal transduction, cell growth and differentiation, regulation of enzyme activities and immune response. Excessive amount of ROS may cause irreversible oxidative damage to DNA, proteins and lipids leading to cell death. ROS-inducing reagents are considered as chemotherapy reagents [20]. We found that the treatment with AQHAR led to induction of ROS in U2OS cancer cells and may cause ROS-mediated selective DNA and mitochondral damage and apoptosis, as reported for some other plant extracts and phytochemicals [21, 22].

**Fig 6:**
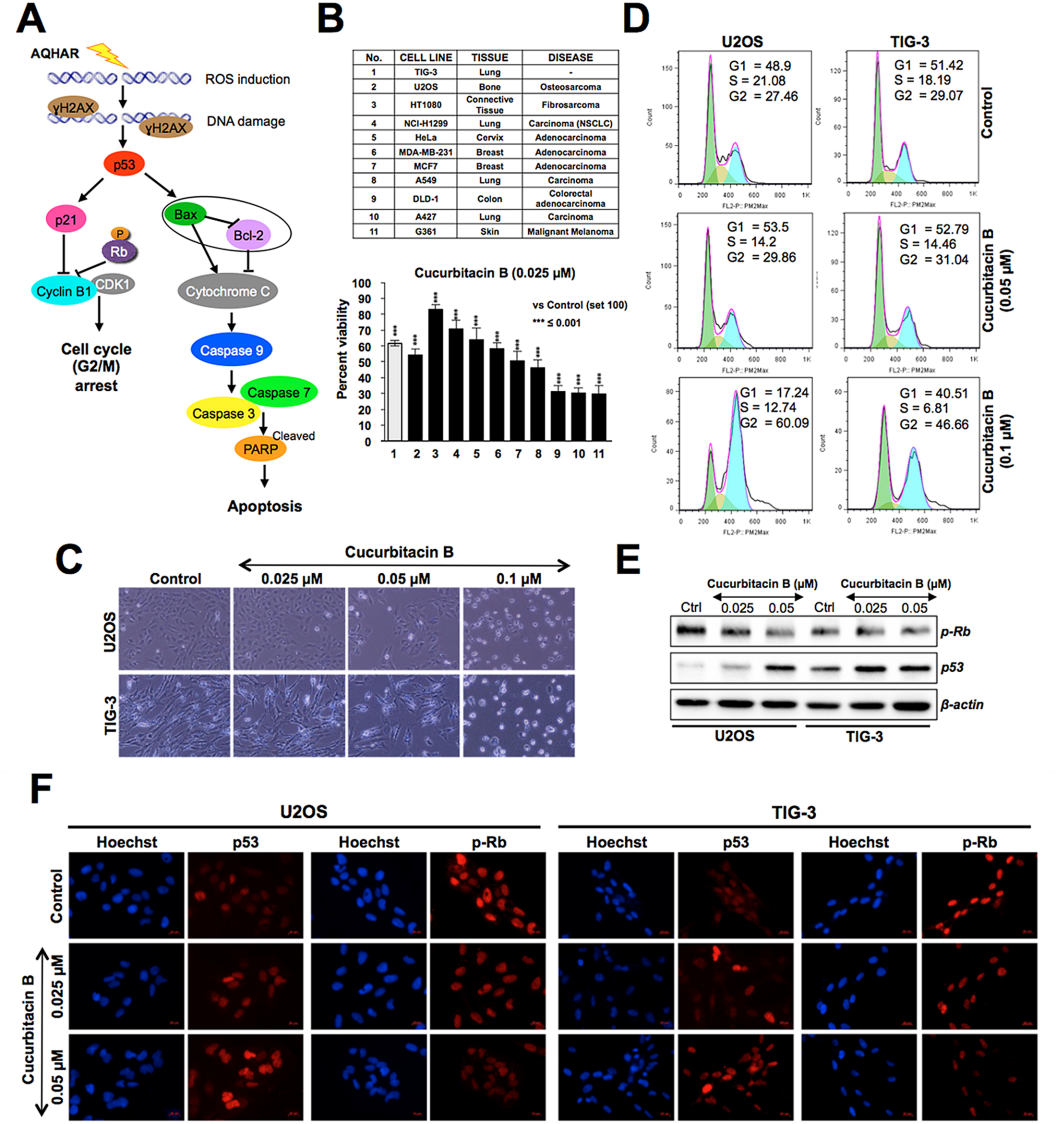
Cytotoxicity of curcubitacin B on cancer and normal human cells. (A) Schematic representation showing the effect of AQHAR on cancer cells by induction of oxidative stress (ROS) and DNA-damage leading to activation of growth arrest and apoptosis signaling. (B) Effect of curcubitacin B on a variety of human cancer and normal cells, ***p<0.001 denotes statistically significant difference between the control and treated groups. (C) Phase contrast images of control and curcubitacin B-treated human osteosarcoma (U2OS) and normal (TIG-3) cells showing the toxicity to both. (D) Cell cycle analysis showing the arrest of U2OS and TIG-3 cells in G2/M phase in response to curcubitacin B treatment. (E) Cucurbitacin B-treated U2OS cells show decrease in phosphorylated RB (pRB) and increase in p53 at doses as low as 0.025 μM. Similar effects were observed by immunostaining in both cancer (U2OS) and normal (TIG-3) cells (F).

Based on the results, it was predicted that the AQHAR-induced selective cytotoxicity to U2OS cells involved activation of DNA damage, p53-p21 and ROS signalings pathways resulting in G2/M phase arrest or caspase-mediated apoptosis (Fig 6A). We next considered that AQHAR may contain cucurbitacin B, a known phytochemical present in alcoholic extracts of *H*. *angustifolia* roots [11], and investigated its selective cytotoxicity to cancer cells. As shown in Fig 6B, cucurbitacin B showed cytotoxicity to a variety of human cancer cells at doses as low as 0.025 to 0.1 μM. We found that some cancer (HT1080, NCl-H1299, HeLa, MDA-MB-231) cells showed poor response to cucurbitacin B, however it was cytotoxic to normal cells (TIG-3) at dose as low as 0.025 μM (Fig 6B and 6C). The data was also supported by direct observation of cells (Fig 6C) and cell cycle analysis (Fig 6D) that revealed that cucurbitacin B caused toxicity to normal cells (human skin fibroblasts). Unlike AQHAR-treated cells, cucurbitacin B-treated cells showed induction of cell cycle arrest in G2/M phase in both U2OS and TIG-3 cells (Fig 6D). Cytotoxicity of cucurbitacin B to human cancer cells has been earlier attributed to disruption of microtubule polymerization [23, 24]. In addition to these reports, we found that the cytotoxicity of cucurbitacin B was mediated by activation of p53 and pRb tumor suppressors (Fig 6E). Furthermore, both Western blotting and immunostaining analyses revealed activation of pRB (decreased phosphorylation) and p53 (increased expression level) in AQHAR-treated TIG-3 cells at doses as low as 0.05 and 0.025 μM, respectively (Fig 6E and 6F). These data suggested that the selective cytotoxicity of AQHAR to cancer cells is caused by components other than cucurbitacin B and there may be or AQHAR may contain some other components that protected normal cells against its toxicity.

### AQHAR caused tumor-suppression and inhibited lung metastasis *in vivo*

**Fig 7:**
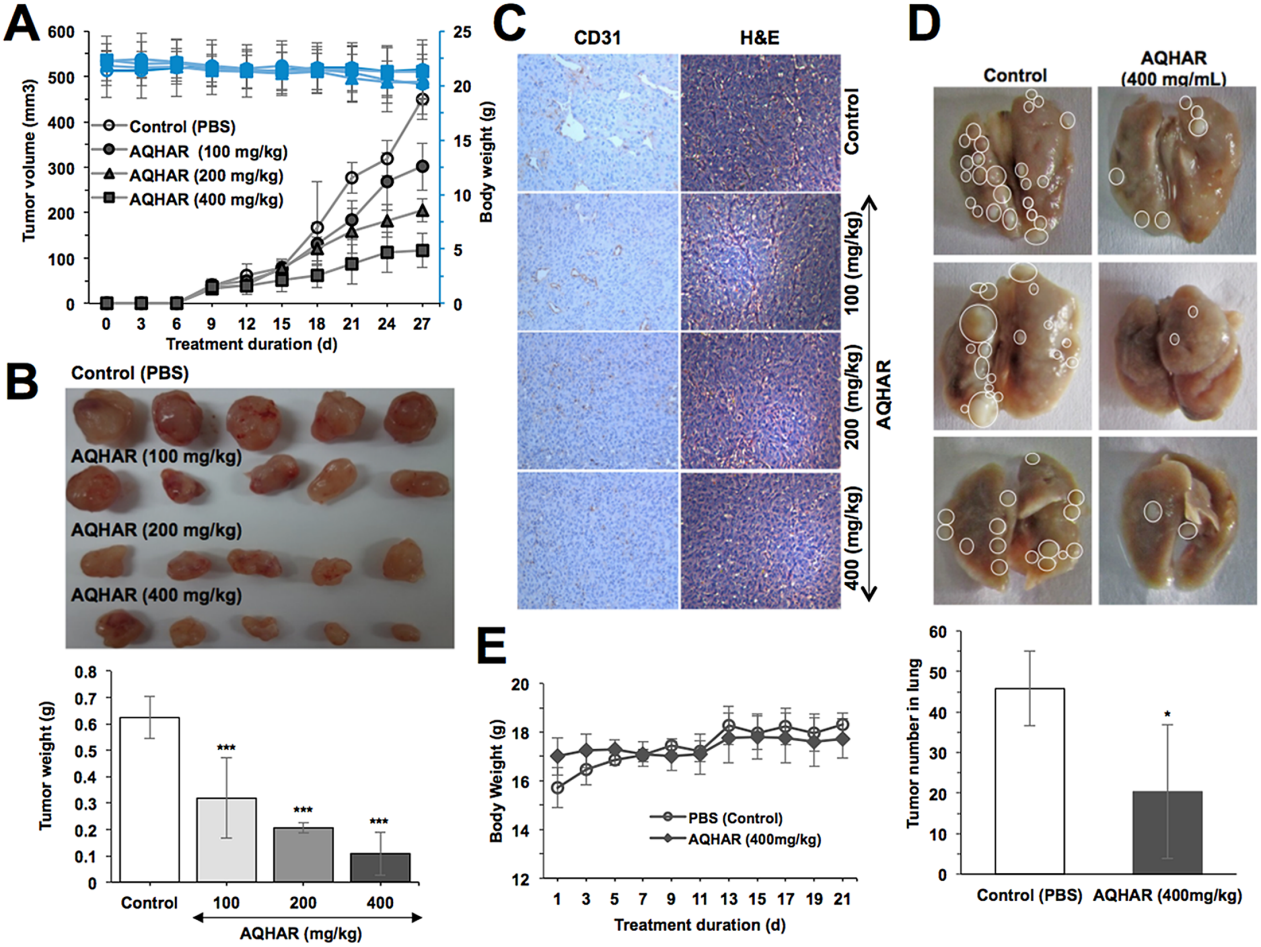
Tumor-suppression by AQHAR treatment in nude mice tumor xenograft assays. (A) Body weight changes and tumor volume in control and AQHAR-fed mice. (B) Images of dissected tumors and their average weight in control and AQHAR-treated groups. (C) Anti-angiogenic effect of AQHAR in HT1080 xenograft tumors as determined by immunohistochemical staining for CD 31, and hematoxylin and eosin. (D) Effect of AQHAR on lung metastasis showing decreased in number of lung tumors in AQHAR-treated group. (E) Change in body weight during tail vein lung metastasis experiments. *p<0.05, **p<0.01 and ***p<0.001 denote the statistically significance between the control and treated groups.

Since the anticancer activity in aqueous extract could be extremely beneficial for human consumption, we examined its effect on *in vivo* tumor progression in nude mice. HT1080 subcutaneous xenograft bearing-mice were fed with AQHAR for 21 days. We found that the tumor volume of control mice gradually increased and at the end of experiment, the average volume of tumors in vehicle group reached to 449.86 ± 44.77 mm^3^. Notably, the average tumor volume in AQHAR-treated mice were only 301.51 ± 52.30, 205.17 ± 25.71 and 116.54 ± 37.17 mm^3^ at the dose of 100, 200 and 400 mg/kg body weight, respectively (Fig 7A). Moreover, at the end of the experiment, tumor tissues were excised from each of the sacrificed mice and weighed (Fig 7B). These data endorsed that the treatment with AQHAR caused significant (*p*<0.001) inhibition of tumor progression in a dose dependent manner. The tumor inhibitory rate was 50.17 ± 18.62%, 66.73 ± 3.48%, and 82.93 ± 11.26% when the mice were fed with AQHAR at the concentration 100, 200 and 400 mg/kg body weight, respectively.

In order to verify the mechanism of tumor suppression by AQHAR, tumors were subjected to histological examination. As seen in Fig 7C, a marked dose-dependent decrease in the number and size of CD31-positive vessels was observed in AQHAR-treated tumors. H&E staining results revealed contrasting results. Whereas tumor mass treated with different doses of AQHAR displayed a large area of necrosis, there was a large area of proliferating tumor cells in the vehicle group.

We also investigated the anti-metastasis activity of AQHAR in nude mice lung metastasis assay. As shown in Fig 7D, control mice showed big tumors with an average tumor number (45.8 ± 9.2) in the lung, whereas AQHAR-treated mice showed significant reduction in the number (20.4 ± 16.5, *p*<0.05) and volume of lung tumors, suggesting AQHAR possessed a strong anti-metastasis activity.

**Fig 8:**
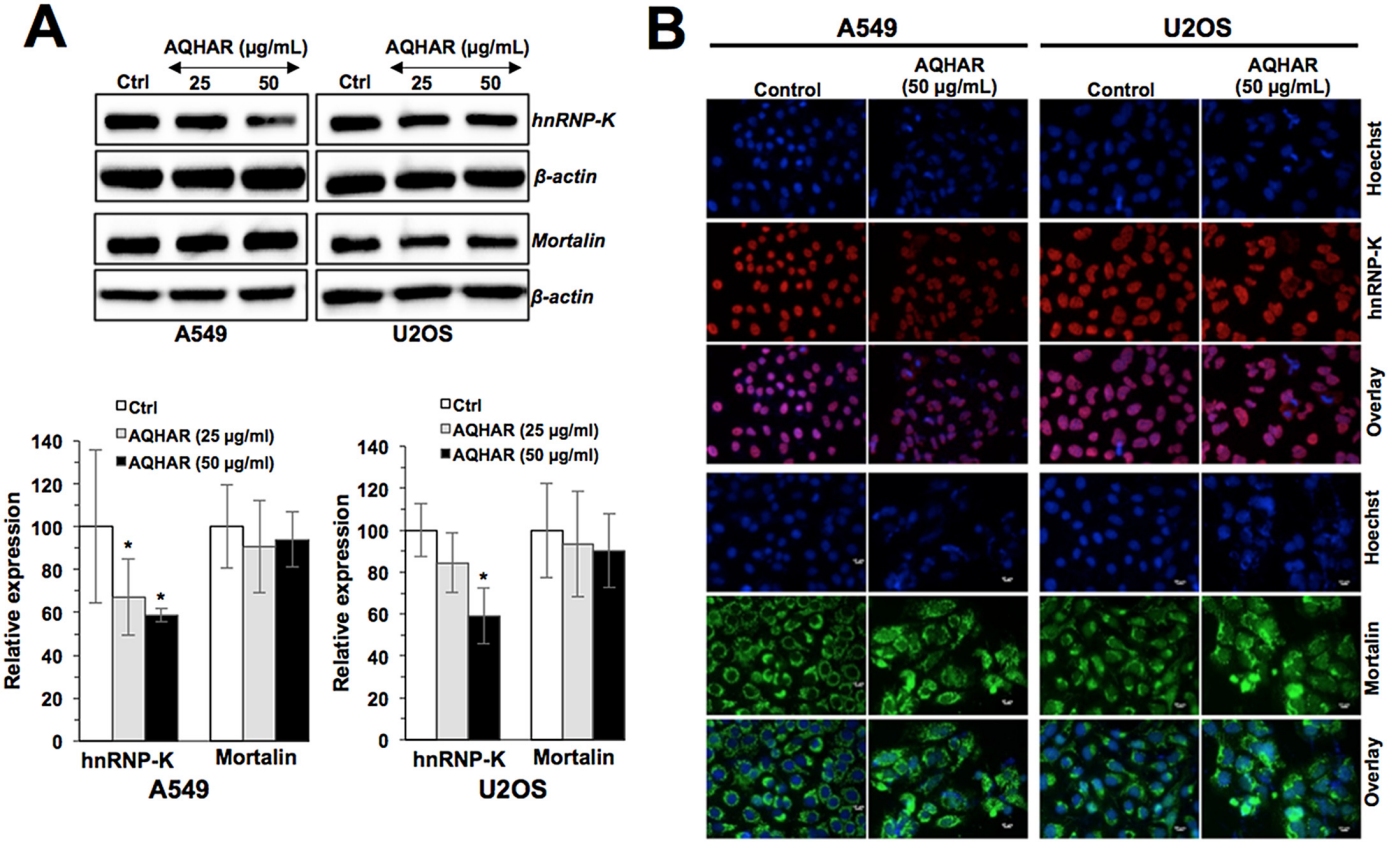
Effect of AQHAR on hnRNP-K and mortalin, tumor metastasis mediating proteins, in A549 and U2OS cells. (A) Western blot analysis for hnRNP-K and mortalin showing decrease in hnRNP-K. (B) Immunostaining of showing decrease in nuclear hnRNP-K and clustering of mortalin staining. Results were represented as mean ± SD of three independent experiments. *p<0.05 denotes the statistically significant difference between the treated and control groups.

We next examined the expression level of metastasis related proteins, hnRNP-K and mortalin in bone (U2OS) and lung (A549) cancer cells in response to AQHAR treatment. It caused downregulation of hnRNP-K expression in a dose-dependent manner (Fig 8A). The results from immunofluorescence analysis also confirmed the downregulation of hnRNP-K in AQHAR-treated both U2OS and A549 cells (Fig 8B). Overexpression of hnRNP-K has been identified as a key regulator of metastasis [25, 26]. Thus, the suppression of hnRNP-K supported anti-metastasis activity of AQHAR. Since mortalin is a protein known to be involved in carcinogenesis and metastasis, we examined the effect of AQHAR on its level and staining pattern [27, 28]. Although no significant difference was found in the level of mortalin expression (Fig 8A), the immunostaining analysis revealed its aggregation in AQHAR-treated cells (Fig 8B) suggesting disruption of its functions including mitochondrial import, chaperoning, intracellular trafficking of other proteins and inactivation of tumor suppressor p53 [27, 28]. Indeed, an activation of p53 was observed in AQHAR-treated cells (Fig 4). Taken together, these data suggested that the treatment with AQHAR (i) caused anti-tumor and anti-metastasis effects, mediated by activation of p53 and inactivation of hnRNP-K signaling and (ii) may involve multiple cellular responds, including apoptosis, growth arrest and induction of senescence in cancer cells.

### AQHAR was safe for mice and induced antioxidant enzyme activities

The toxicity in mice was monitored by feeding them with AQHAR and keeping records of their activity and body weight throughout the experiment. No toxicity was observed with any of the doses used in the present study (Fig 7A and 7E). In order to further investigate the cytotoxic effect of AQHAR administration to the host, the levels of AST and GPT (markers of liver function [29]) were measured in control and AQHAR_-_treated mice groups, along with the plasma level of TP and GLU. Results of these biochemical parameters showed no signs of liver damage or any cytotoxic effect to the host (Table 1). Decrease in the activities of antioxidant enzymes such as SOD and CAT in the blood has been correlated with malignant transformation [30]. The increase of lipid peroxidation in the blood, represented by the amount of MDA, signals the organ injury that is also associated with cancer [31]. We examined the levels of SOD, CAT and MDA levels in control and AQHAR-treated mice. As shown in Table 1, AQHAR-treated mice possessed an elevated level of SOD (with 100, 200 and 400 mg/mL, *p*<0.05) and CAT (with 200 and 400 mg/mL, *p*<0.01), and reduced level of the MDA level (with 400 μg/mL, *p*<0.05) suggesting that in addition to the activation of DNA-damage response and p53-p21 signaling in cancer cells, AQHAR activated antioxidant signaling in mice that may adds to its anticancer activity. Taken together, we propose that AQHAR could be useful as a potent and safe natural anticancer medicine.

**Table 1:** Effect of AQHAR on antioxidant enzyme activities and biochemical parameters in mice blood.

Parameters	Control (PBS)	AQHAR (100 mg/kg)	AQHAR (200 mg/kg)	AQHAR (400 mg/kg)
SOD (U/mg protein)	89.40 ± 6.24	106.14 ± 2.83*	128.45 ± 12.5***	180.14 ± 9.48***
CAT (U/mg protein)	0.18 ± 0.01	0.20 ± 0.01	0.21 ± 0.01**	0.24 ± 0.02***
MDA (μmol/L)	1.71 ± 0.65	1.13 ± 0.06	0.88 ± 0.06	0.71 ± 0.06*
TP (mg/mL)	4.48 ± 0.61	4.98 ± 0.38	4.64 ± 0.38	4.90 ± 0.62
GLU (g/L)	251.0 ± 71.96	248.33 ± 74.54	244.0 ± 47.08	232.33 ± 51.93
AST (U/L)	109.75 ± 26.59	116.25 ± 57.72	121.25 ± 47.12	104.50 ± 25.09
ALT (U/L)	27.25 ± 9.81	21.50 ± 7.05	29.00 ± 10.89	28.75 ± 16.82

Data are expressed as mean ± SD (n = 5).

*p<0.05,

**p<0.01

***p<0.001 denote the statistically significant difference between AQHAR-treated and control groups.

SOD, superoxide dismutase; CAT, catalase; MDA, malondialdehyde; AST, aspartate aminotransferase; ALT, alanine aminotransferase; TP, total protein; GLU, glucose.

Herbal medicine has been known to enhance the immune function, speed up recovery, alleviate radiochemotherapy-related toxicities, improve quality of life, and extend survival and hence has been popular as complementary treatments [32]. However, the exact mechanism of their activties has not been resolved and limits their usage in clinic. We, hereby, report that the aqueous extract of *H*. *angustifolia*, (AQHAR) causes selective toxicity to cancer cells. The mechanism is through an induction of cell cycle arrest at G2 phase, caspases-mediated apoptosis and activating the DNA damage as well as oxidative stress responses (Figs 4 and 5). Of note, normal cells remained unaffected at the equivalent doses. Moreover, the selective cancer cell cytotoxicity was translated *in vivo* by tumor growth suppression, anti-angiogenesis and anti-metastasis in mice and without any apparent toxic effect to them (Figs 7 and 8). In light of the data that both cell cycle arrest and apoptosis were observed in AQHAR-treated cells, it is likely that the cancer cells, in different cell cycle stages, respond in different ways and that attributes to the anti-metastasis and anti-angiogenic activities as observed in *in vivo* assays (Fig 7). Further studies are warranted to dissect the molecular mechanisms of such multiple activties of AQHAR followed by its possible recruitment for effective cancer treatment.

### Conclusion

The present study provides, for the first time, the molecular evidence of anticancer activity in the aqueous extract of *H*. *angustifolia* roots (AQHAR) *in vitro* and *in vivo*. AQHAR is proposed as a promising natural and safe anticancer reagent for cancer treatment, and warrant further studies.

## Materials and Methods

### Plant collection and preparation of the aqueous extract of *H*. *a**ngustifolia* roots (AQHAR)

The roots of *H*. *angustifolia* used in the present study were collected from Vientianei, Laos (18.069609, 102.828844) in June 2013. Botanical authentication was performed at the herbarium of Kunming Institute of Botany, Chinese Academy of Sciences along with the deposition of a voucher specimen (No. 090155).

The dried roots of *H*. *angustifolia* were grounded into fine powder (particle size <0.6 mm), extracted with distilled water at room temperature for 24 h (twice) to obtain 8.4% extract. The filtrates of two extractions were combined, concentrated with a rotary vacuum evaporator at 50°C, lyophilized and stored at -20°C until further use.

### Cell lines and culture

Human cancer and normal cells, obtained from the Japanese Collection of Research Bioresources (JCRB, Japan), were maintained in Dulbecco’s modified Eagle’s minimal medium (DMEM; Invitrogen)-supplemented with 10% FBS and 1% penicillin/streptomycin at 37°C in a humidified incubator with 5% CO_2_.

### Cytotoxic/cell growth inhibition assay and morphological observations

The cytotoxic effect of AQHAR was determined by MTT {3-(4, 5- dimethylthiazol-2-yl)-2, 5-diphenyltetrazolium bromide (Life Technologies)} assay as described earlier [33].

For morphological observation, cells were seeded at 2 X 10^5^ cells/well in 6-well plates, and allowed to adhere for approximately 24 h following which the culture medium were replaced with DMEM medium containing 0, 25, or 50 μg/mL of AQHAR, respectively. After 24 h of incubation, the morphology of cells was directly recorded using a phase-contrast inverted microscope fitted with digital camera (Digital sight DS-L1, Nikon, Japan).

### Colony forming assay

Effect of AQHAR on long-term proliferation of cancer cells was determined by colony forming assay. 5 X 10^2^ cells/well were seeded in a 6-well dish, allowed to adhere for approximately 24 h, and treated with DMEM containing AQHAR (6.25, 12.5, 25 or 50 μg/mL) for 6 h. The treated cells were maintained in normal medium until the appearance of colonies. The culture medium was replaced with fresh medium every third day. Cells were fixed, stained, photographed and counted as described earlier [34].

### Detection of reactive oxygen species (ROS)

The ROS were detected by fluorescent staining using the Image-iT^TM^ LIVE Green Reactive Oxygen Species Detection Kit (Molecular Probes Inc, USA), following the procedure recommended by the manufacturers and as described earlier [33]. Cells were treated with AQHAR (50 μg/mL) for 24 h and counter stained with Hoechst 33342 as described earlier [35].

### Single cell gel electrophoresis assay (Neutral comet assay)

Neutral comet assay was performed using Trevigen’s CometAssay^®^ Electrophoresis System following the protocols recommended by the manufacturer. Briefly, U2OS and TIG-3 cells were seeded (2 × 10^5^ cells/well) in 6-well plates, and treated with or without 25 μg/mL of AQHAR (37°C, 24 h) or 100 μM H_2_O_2_ (4°C, 20 min, positive control). After the treatments, cells were collected, centrifuged (1,500 rpm, 3 min), washed and re-suspended in cold PBS. Then 5 X10^3^ cells were combined with 500 μL of molten low melting agarose (at 37°C) and immediately pipetted 50 μL of the mixture onto a well CometSlide^™^ with care. After being kept at 4°C in dark for 10 min, the slides were immersed in pre-chilled lysing solution for 1 h and the neutral electrophoresis buffer for 30 min at 4°C, respectively. The electrophoresis (21 V, 30 min) was applied in the same buffer at 4°C. The slides were then immersed in DNA precipitation solution and 70% ethanol successively for 30 min each time. After 30 min of staining of SYBR^®^ Green at room temperature in dark, DNA damage and migrating fragments (comet tail) was observed under Carl Zeiss microscope with epifluorescence optics. Quantitative analysis was determined by using CASP software, and 50 randomly selected cells were analyzed per sample.

### Cell cycle analysis

U2OS and TIG-3 cells were seeded at a density of 2 X 10^5^ cells/well in 6-well plates. After 24 h of seeding, cells were treated with different concentrations of AQHAR (0, 25 and 50 μg/mL) for 24 h followed by harvesting by trypsin. The cell pellets were fixed with ice-cold 70% ethanol and stored at -20°C until further use. The fixed cells were centrifuged at 500x*g* for 5 min, washed twice with cold PBS (1 mL) and re-suspended in 0.25 mL PBS. To avoid false DNA-PI staining, RNA was removed by adding 5 μL of RNase A (10 mg/mL) at 37°C for 1 h followed by centrifugation (500x*g*) for 5 min. Supernatant was replaced by 200 μL of Guava Cell Cycle reagent and incubated in dark for 30 min. The stained cells were subjected to cell cycle analysis using Guava PCA flow cytometer (Millipore) and FlowJo Software (version 7.6, Flow Jo, LLC, USA).

### Apoptosis assay

U2OS cells were seeded at a density of 2 X 10^5^ cells/well in 6-well plates and cultured in medium for 24 h, followed by AQHAR treatments (0, 25 and 50 μg/mL). Apoptosis assay was examined using Guava Nexin Reagents following the procedure recommended by the manufacturer, and as described earlier [34]. Number (%) of apoptotic cells was determined by FlowJo Software.

### Western blotting

The expression levels of proteins were determined by Western blotting as described earlier [26]. Immunoassays were performed with anti-p53 (DO-1), -p21 (C-19), -caspase 7 (C-18), -caspase 9 (H-83), -Bcl-2 (N-19), -Bax (N-20), -PARP (H-250) (Santa Cruz Biotechnology, Inc.), anti-cyclin B1, -caspase 3 (BD biosciences), anti-hnRNP-K (ImmuQuest), anti-pRb (ser-780), anti-actin (Chemicon International, CA) and anti-mortalin antibodies [33]. Quantitation of immunoblots was performed using the Image J software (National Institute of Health).

### Immunocytochemistry

Cells were seeded on 18-mm glass coverslips at 1 × 10^5^ cells/well in 12-well plates, and treated with 50 μg/mL of AQHAR for 24 h. Immunostaining was performed using antibodies including, anti -p53 (DO-1), -p21 (C-19), -cyclin B1, -phospho- histone-γH2AX (S780) (Cell Signaling Technology), and anti-hnRNP-K and protocols described earlier [35]. The cells were examined under Carl Zeiss microscope with epifluorescence optics.

### Animals

Five-week old female BALB/c nude mice were purchased from CLER Japan, Inc. (Tokyo, Japan) for subcutaneous xenograft experiments and maintained in Laboratory Animal Resource Center at the University of Tsukuba (Tsukuba, Japan). For tail vein- lung metastasis experiment, four-week old female BALB/c nude mice were bought from Beijing HFK Bioscience Co., Ltd (Beijing, China) and maintained in the Institute of Laboratory Animal Sciences of Chinese Academy of Medical Sciences. All mice were kept in pathogen-free autoclaved cages and maintained under controlled conditions with temperature of 23 ± 1°C, humidity of 55 ± 5% and a 12 h light/dark cycle. They were fed with standard rodent chow and water *ad libitum*. Mice were allowed to acclimate to the housing conditions for one week prior to experimentation. All the animal experiments were carried out in strict ethical accordance, following the recommendations by the Animal Experiments Committee, University of Tsukuba (Approval No. 14–375) and institutional regulations of Chinese Academy of Medical Sciences (Approval No. ILAS-PG-2014-018), respectively.

### Tumor suppression assay

For subcutaneous xenograft model, HT1080 cells (0.2 mL, 1 × 10^7^ cells) were injected subcutaneously into the inguinal area of each mouse. Twenty mice were randomly divided into 4 groups after 6 days; Group 1 (positive control, 0.2 mL/d, PBS), Group 2 (0.2 mL/d, 100 mg/kg of AQHAR diluted in PBS), Group 3 (0.2 mL/d, 200 mg/kg of AQHAR diluted in PBS), and Group 4 (0.2 mL/d, 400 mg/kg of AQHAR diluted in PBS). The animals were orally administrated with AQHAR or vehicle (PBS) for 21 days till tumors reached the targeted volume of 500 mm^3^ in the control group. To monitor the toxicity of this extract, the body weight of each animal was taken every 3 days. Tumor volume in mice was measured and calculated every 3 days according to the formula: length × width^2^ × 1/2 [36]. At the end of the experiment, mice were anaesthetized with isoflurane inhalation and their blood samples were taken from heart and collected in polyethylene tubes. The plasma samples were obtained by centrifugation (10,000 rpm, 4°C) for 10 min and kept frozen at -80°C until further use as described below. Mice were sacrificed at the end of the treatment and the tumor tissues were removed and weighed. The tumor inhibitory rates were calculated by using the following formula: Tumor inhibitory rate (%) = (Wc—We) x 100/Wc (*Wc* is the mean tumor weight of the control mice, and *We* is the mean weight of the treated mice).

For lung metastasis model, HT1080 cells (0.2 mL, 1 × 10^6^ cells/mice) were injected into the nude mice through tail vein. Ten mice were randomly divided into 2 groups; Group 1 (positive control, 0.2 mL/d, PBS) and Group 2 (0.2 mL/d, 400 mg/kg of AQHAR diluted in PBS). The animals were orally administrated with AQHAR or vehicle (PBS) for 21 days and monitored their body weight every alternate day. At the end of the experiment, the mice were sacrificed and their lungs were excised, removed and evaluated for the presence of tumors.

### Evaluation of tumor xenograft by histology and immunohistochemistry

Tumor tissues were fixed with formaldehyde, embedded in paraffin, and sectioned into 3-mm thick sections. Representative sections were stained with hemotoxylin and eosin (H&E) and examined by light microscopy. To detect the microvessel density, slides were deparaffinized in xylene and processed for CD31 staining (PECAM-1, Cell Signaling Technology) as described previously [27].

### Measurement of biochemical parameters in blood

The antioxidant enzyme activities of superoxide dismutase (SOD) and catalase (CAT) along with malondialdehyde (MDA) level in the plasma were measured using the corresponding assay kits according to the protocols recommended by the manufacturer, respectively. Superoxide dismutase (SOD) Assay Kit (Cat no. S311) was purchased from Dojindo Molecular Technologies, Japan, Colorimetric Activity kit (CAT) (Cat no. K033-H) was obtained from Arbor Assays, USA, and 2-Thiobarbituric Acid Reactive Substances (TBARS) Kit (Cat no. FR40) was from Oxford Biomedical Research, USA. Plasma levels of aspartate aminotransferase (AST), alanine aminotransferase (ALT), total protein (TP) and total glucose (GLU) were analyzed using an automated biochemistry analyzer (FUJI DRI-CHEM 7000, Japan).

### Statistical analysis

All experiments were carried out in triplicates, and data were expressed as mean ± standard deviation (SD). Statistical analysis was performed using the SPSS 13.0 software (SPSS Inc., Chicago, USA). Differences among samples were evaluated by using analysis of variance (ANOVA) and Duncan’s multiple comparison method. Significant difference was assumed at *p*<0.05.
